# Intra- and interbrain synchronization and network properties when playing guitar in duets

**DOI:** 10.3389/fnhum.2012.00312

**Published:** 2012-11-29

**Authors:** Johanna Sänger, Viktor Müller, Ulman Lindenberger

**Affiliations:** Center for Lifespan Psychology, Max Planck Institute for Human DevelopmentBerlin, Germany

**Keywords:** functional connectivity, graph theory, EEG hyperscanning, joint action, cortical phase synchronization, social interaction, music

## Abstract

To further test and explore the hypothesis that synchronous oscillatory brain activity supports interpersonally coordinated behavior during dyadic music performance, we simultaneously recorded the electroencephalogram (EEG) from the brains of each of 12 guitar duets repeatedly playing a modified Rondo in two voices by C.G. Scheidler. Indicators of phase locking and of within-brain and between-brain phase coherence were obtained from complex time-frequency signals based on the Gabor transform. Analyses were restricted to the delta (1–4 Hz) and theta (4–8 Hz) frequency bands. We found that phase locking as well as within-brain and between-brain phase-coherence connection strengths were enhanced at frontal and central electrodes during periods that put particularly high demands on musical coordination. Phase locking was modulated in relation to the experimentally assigned musical roles of leader and follower, corroborating the functional significance of synchronous oscillations in dyadic music performance. Graph theory analyses revealed within-brain and hyperbrain networks with small-worldness properties that were enhanced during musical coordination periods, and community structures encompassing electrodes from both brains (hyperbrain modules). We conclude that brain mechanisms indexed by phase locking, phase coherence, and structural properties of within-brain and hyperbrain networks support interpersonal action coordination (IAC).

## Introduction

Social interaction is an ubiquitous ingredient of human life; our minds and brains function and are formed in interaction with other people (Hari and Kujala, 2009). Coordinating one's behavior with that of an interaction partner requires the perception, representation, and anticipation of both one's own and the partner's actions (e.g., Pecenka and Keller, 2011). Recently, we have proposed that the coordination and partial integration of two or more forward models of action control (Wolpert et al., 1995) into a joint, interpersonally shared forward model may help to initiate and sustain interpersonal action coordination (IAC; see Sänger et al., 2011). This process is likely to engage the mirror neuron system (Blakemore and Decety, 2001; Rizzolatti et al., 2001; Pacherie and Dokic, 2006; Gallagher, 2009). In addition to identifying the brain regions supporting IAC (for a review see Sänger et al., 2011), it seems worthwhile to explore and identify neural codes that support the representation of joint action. Here, coherent brain oscillations may play a pivotal role, especially in tasks that require the close alignment (coordination) of one's own and the other's action in real time. This hypothesis is consistent with available evidence about the functional significance of brain oscillations in perception and action (Sanes and Donaghue, 1993; Makeig and Jung, 1996; Kilner et al., 2000). From a more general perspective, coherent brain oscillations allow for fast and precise information exchange (Roelfsema et al., 1997) and bind neuronal information from different regions (Varela et al., 2001), thereby qualifying as candidate brain mechanism of interpersonally coordinated behavior and social interaction.

So far, only a few studies have investigated this assumption by taking simultaneous neuroelectrical recordings of multiple interacting individuals (cf. Hasson et al., 2012). Dumas et al. (2010) observed coherent brain oscillations between electrodes of the model and the imitator during behaviorally synchronous sequences of a gestural imitation task. Cui et al. (2012) reported coherence between NIRS time-series obtained from simultaneous measurement of dyads engaged in a digital game of cooperation and competition. Coherence was only found during cooperation, not competition, and occurred in the superior frontal cortex, which is implicated with modeling and predicting the actions of others. Yun et al. (2008) revealed synchronized high-frequency oscillations in right fronto-central regions of dyads engaged in the Ultimatum Game. By estimating nonlinear dependencies between the two EEG time series, they showed information flow from the responder's left fronto-central region to the perceiver's right homologue, and concluded that this region may play a prominent role in social decision making. Astolfi et al. (2010) simultaneously collected EEG data from seven groups of four people each, who were playing cards among one another. They found functional connectivity between signals of players from the same team, suggesting that players only showed interrelated brain activity if they had some interest in coordinating their behavior with each other.

The present study builds directly on an earlier investigation by Lindenberger and colleagues (2009). Investigating guitar duets playing in unison, the authors found increased phase synchronization within and between the guitarists' brains during periods of preparatory metronome tempo setting and at the onset of coordinated play. These couplings were primarily observed in the delta and theta frequency ranges and at frontal as well as central electrodes. Lindenberger et al. also found that the intrabrain phase alignment was strongly related to the degree of behavioral play-onset synchrony between the two guitarists of a pair on a given trial. The latter result suggests that the degree of phase synchronization does, in fact, reflect the dynamics of behavioral interaction between the guitarists.

In light of the promising findings by Lindenberger et al. (2009), the hypothesis that within- and between brain neural couplings represent a mechanism for IAC merits further scrutiny. Of special importance are attempts to rule out alternative hypotheses. For instance, the sheer similarity of perceptual input and performed action between two individuals engaged in joint action may be sufficient to induce interbrain coherence without serving a functional role in IAC. As an attempt to better disambiguate the two hypotheses, the present study went beyond Lindenberger et al. (2009) by introducing a more complex piece of music in two voices, such that the two guitarists would not play exactly the same tune. Furthermore, we experimentally manipulated the musical roles of leader and follower to look at asymmetries in oscillatory correlates of IAC. Assigning such social roles in musical performance manipulates coordination demands while leaving most perceptual or motor aspects of the situation untouched.

The specific assumptions and research goals of this study can be summarized as follows. First, we wished to replicate the finding reported by Lindenberger et al. (2009) of fronto-central synchronization in low frequency bands during preparatory tempo setting and coordinated play onset with guitar duets playing in two voices, that is, when the two guitarists of a duet do not play exactly the same tune. Low frequencies are regarded as relevant since they have previously been implicated in social coordination (Tognoli et al., 2007), interpersonally shared task representation (Sebanz et al., 2006), motoric functions (Andres et al., 1999; Kilner et al., 2000; Deiber et al., 2001; Grosse et al., 2002; Waldert et al., 2008) and sensorimotor integration (Caplan et al., 2003). We expected frontal and central electrodes to be predominantly involved, as they cover the prefrontal cortex, which has been associated with Theory of Mind activity (Rizzolatti et al., 2001; Gallagher and Frith, 2003; Dziobek et al., 2011), the premotor cortex, where the human mirror neuron system is suspected (Rizzolatti, 2005), and the motor and somatosensory cortices, which regulate motor control (cf. Novembre et al., 2012) and are also activated during music production (Zatorre et al., 2007).

Second, we intended to back up the assumption that synchronized brain oscillations are a correlate of IAC and not just a by-product of shared perceptions and similarity in movements. Therefore, we experimentally manipulated the musical roles of leader and follower. Specifically, the leader had to bring the other one in and keep time, while the follower had to heed to the tempo-induced by the leader. We hypothesized that these two complementary roles would be reflected in asymmetric patterns of cortical phase synchronization. In addition, and informed by the Lindenberger et al. (2009) findings, we also compared segments of coordinated play onset with segments of mere joint playing. We expected greater synchronization at coordination points even though the degree of similarity in perception and action between the two players would be about the same for coordination points and joint playing of other parts of the musical score.

Third, we were interested in the functional interbrain networks that would emerge within and between the brains of duet partners in the delta and theta frequency ranges. Similar to Babiloni and colleagues (2007a,b), Astolfi et al. (2010) and De Vico Fallani et al. (2010), network properties were explored by submitting the phase coherence measures to a graph analysis of intra- and interbrain phase coherence (IPC). Again, we assumed that frontal and central sites would emerge as particularly relevant, especially during musical coordination points. Going beyond the previous studies, our analyses were not restricted to connectivity strengths, but also aimed at understanding additional network properties. In particular, we expected that the small-world properties of within-brain and hyperbrain networks, as indexed by the simultaneous presence of functional integration and segregation (Sporns and Zwi, 2004), would be enhanced during periods of increased demand for musical coordination. Small-worldness can be found in various kinds of networks (Sporns and Zwi, 2004) and might reflect an optimal architecture for information processing (Stam, 2004). Finally, we expected that the hyperbrain network would show a non-random community structure containing hyperbrain modules, that is, groups of strongly interconnected electrodes that do not belong to the same brain.

## Materials and methods

### Research participants

Thirty-two guitarists participated in the study, forming a total of 16 non-overlapping duets. Four of these duets had to be excluded from analysis since they provided less than 30 trials in which EEG data during the relevant segments was artifact free. In seven out of the twelve remaining pairs, both partners were male; four duets were mixed, and only one duet had two female players. The age of the participants ranged from 20 to 58 years (*M* = 35.58, SD = 1.82). Participants had been playing guitar for 22.92 years on average (SD = 11.64). Twenty-two of them played more than once a week, only two played less. Fifteen were currently playing in a musical ensemble, seven had been members of an ensemble before. Ten had studied or were studying music at a conservatory. All participants volunteered for the experiment, and gave their written informed consent prior to their inclusion in the study. The Ethics Committee of the Max Planck Institute for Human Development approved the study. The study was performed in accordance with the ethical standards laid down in the 1964 Declaration of Helsinki.

### Musical material

**Figure 1:**
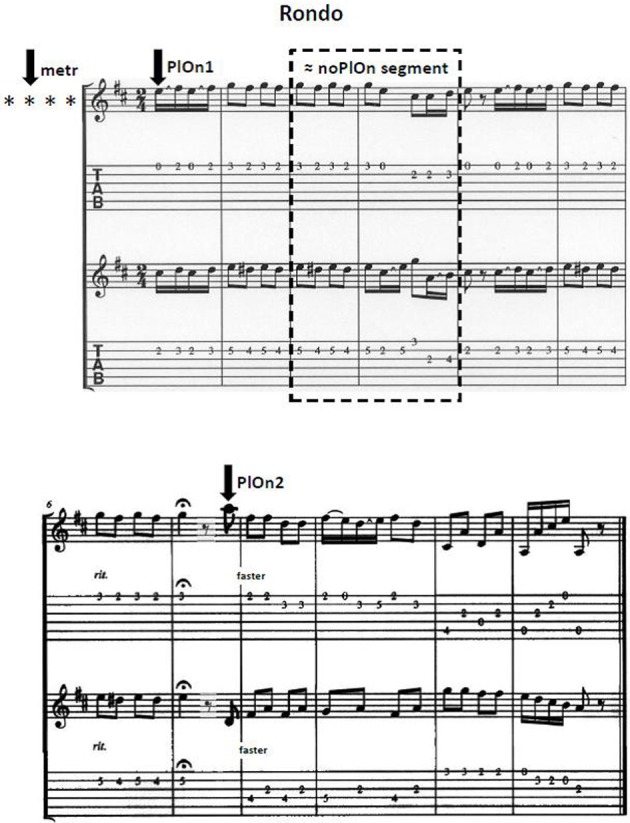
**Note sheet of the adapted version of the Rondo in D-Major by C.G. Scheidler**. Intially, three segments of 3000 ms each were analyzed, each one beginning 1000 ms before the respective stimulus and ending 2000 ms after it. metr: the segment of preparatory tempo setting was time-locked to the second of four metronome beats preceding each trial; PlOn1: the segment around the first play onset was time-locked to the first play onset of the leading guitarist; the segment for the second play onset (PlOn2) was defined accordingly.

The piece of music played during the measurement was an adaptation of a short Rondo sequence from the Sonata in D Major by Christian Gottlieb Scheidler (1752–1815). To avoid the confounding of musical role and voice, we modified the piece such that both voices were as equal as possible instead of constituting a typical leading and typical accompanying voice. Apart from the initial play onset, the piece contained another play onset following a decrease in musical tempo (i.e., *ritardando*), and an eighth rest. After this second play onset, the playing tempo was increased (see Figure 1 for the note sheet). Each participant was given the sheet of music in advance, and was asked to rehearse and memorize one of the two voices, which they then played by heart during the experiment.

### Procedure

Measurement took place in an electromagnetically shielded cabin, in which participants sat face-to-face to each other. Participants were instructed to avoid all unnecessary movement and to execute the picking movements as small as possible in order to virtually avoid movement artifacts. One participant was assigned the leading role, meaning that he or she was responsible for bringing the other in and determining the playing tempo. The follower was asked to exclusively orient himself toward the leader. To account for person variables possibly interfering with this role assignment, we tried to match the partners as far as possible regarding the following aspects: their age, how many years they had already been playing guitar, whether they had studied/were studying at a conservatory, whether they worked as guitarist and whether they currently were a member of a music ensemble. However, we considered these aspects of qualification as complementary, such that a lack in one could be compensated by another one. Please see Appendix A for the pairs at a glance. The Rondo was played a total of 60 times, in two blocks of 30 trials each. Each trial was initiated by four metronome beats (80 bpm), after the last of which the leader signaled the beginning of the play by calmly breathing in. The entire testing session was repeated on another day, with reversed assignment of the leading vs. following role.

### EEG data acquisition

The EEG was recorded with active 64 Ag/AgCl electrodes per person, placed according to the international 10–10 system, with the reference electrode at the right mastoid (actiCAP, Brain Products, Munich, Germany). Separate amplifiers (BrainAmp DC, BrainProducts, Munich, Germany) with separate grounds were used for each individual, linked to one computer. Vertical and horizontal electrooculograms (EOGs) were recorded to control for eye blinks and eye movements. Moreover, an acceleration sensor was applied on each hand of both guitarists to follow the hand movements. Through two microphones, the two guitars were also recorded on one channel each, simultaneous to the EEG recordings. Both hand movement and microphone signals were recorded using a bipolar amplifier (BrainAmp ExG, Brain Products, Munich, Germany). The sound was additionally recorded together with a video of the session, using Video Recorder Software (Brain Products, Munich, Germany) synchronized with the EEG data acquisition. All channels were recorded at a sampling rate of 5000 Hz in order to have a good time and correspondingly frequency resolution for acoustic microphone signals. A 1–1000 Hz bandpass filter was activated. With the help of the audio, video and hand movement recordings, event markers were later set off-line into the EEG data. Markers for the metronome beats were automatically set online during the measurement.

### EEG data analysis

#### Preprocessing

Event triggers were placed for the two play onsets of the Rondo. EEG data were re-referenced offline to an average of the left and right mastoid, resampled at 1000 Hz and then filtered with a band pass ranging from 1 to 70 Hz. Eye movement correction was accomplished by independent component analysis (Vigário, 1997; Jung et al., 1998a). As artifact rejection based on a gradient (a maximum admissible voltage step of 50 μV), and a difference criterion (a maximum admissible absolute difference between two values in a segment of 200 μV) did not render satisfactory results, artifacts from head and body movements were finally rejected by visual inspection only. Spontaneous EEG activity was segmented into epochs of 3 s according to the second metronome beat and the two play onsets of the leading guitarist. Each segment started 1 s before the relevant event and ended 2 s after it. The 12 duets, which provided more than 30 artifact-free trials for all three epochs, on average rendered 54.42 (SD = 10.48) trials for the epoch around the second metronome beat, 54.38 (SD = 12.06) trials for the first and 56.54 (SD = 11.39) for the second play onset.

#### Synchronization measures

Artifact-free epochs were analyzed using a complex Gabor expansion function that transforms the EEG time series into a complex time-frequency signal for frequencies up to 20 Hz. The frequency resolution here was 0.33 Hz and the temporal resolution was 1 ms. Two synchronization measures were obtained from the corresponding time-frequency matrices (Müller et al., 2009): The phase locking index (PLI) reflects the invariance of phases at a single electrode across *k* trials in the time-frequency domain and is defined by

$$\text{PLI}\left(f_{n},\;t\right)=\left|\langle e^{j*\phi^{k\left(f_{n},\;t\right)}}\rangle \right|,\;j=\sqrt{-1}.$$

The intra- and interbrain phase coherence (IPC) represents the degree of constancy in phase difference across *k* trials between two electrodes measured from one or respectively two brains simultaneously. It is defined as

$${\text{IPC}}_{\phi }\left(f_{n},\;t\right)=\left|\langle e^{j*\Delta \phi^{k\left(f_{n},\;t\right)}}\rangle \right|,\;j=\sqrt{-1}$$

with the phase difference

$$\Delta \phi^{k}=mod\left(\phi_{1}^{k}\left(f_{n},\;t\right)-\phi_{2}^{k}\left(f_{n},\;t\right),\;2\;\cdot \;\pi \right)$$

referring to two electrodes, either within one brain in the intrabrain case, or of two brains IPC. This was done for a selection of 21 electrodes per person (Fp1, Fpz, Fp2, F7, F3, Fz, F4, F8, T7, C3, Cz, C4, T8, P7, P3, Pz, P4, P8, O1, Oz, and O2) respectively for all possible pairs of these. This selection reduces a possible bias in functional connectivity findings produced by volume conductance, while still covering the entire cortex, such that the information of the remaining electrodes would be rather redundant.

#### Statistical evaluation of phase-locking values

**Figure 2:**
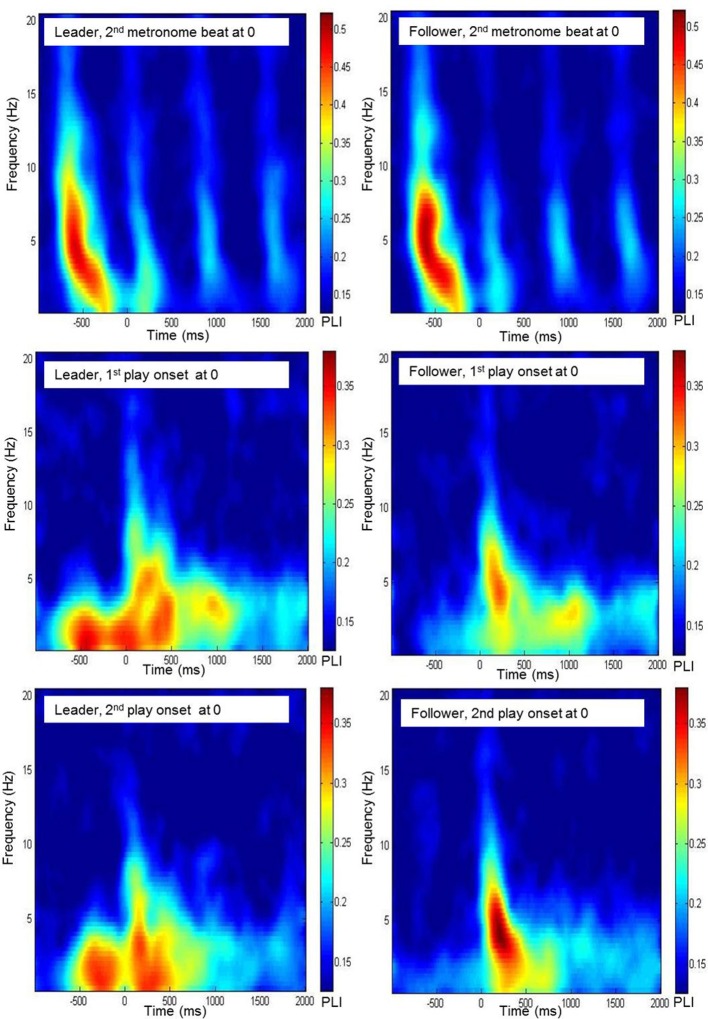
**Time-frequency diagrams of the grand average of the phase locking index, averaged across frontal electrodes (Fp1, Fpz, Fp2, F7, F3, Fz, F4, F8) for leaders and followers during preparatory tempo setting and around coordinated play onsets**.

The significance threshold for PLI values was obtained from surrogate data: After shuffling the time series of each channel, PLI was derived as in the original data. From these values, 1000 bootstrapping samples were drawn. The threshold was then defined as the bootstrapping mean plus three times the bootstrapping standard deviation (*M*_boot_ + 3 × SD_boot_). This procedure yielded a significance threshold of.12. PLI values were averaged within three groups of electrodes: frontal (Fp1, Fpz, Fp2, F7, F3, Fz, F4, and F8), central (T7, C3, Cz, C4, and T8) and parieto-occipital (P7, P3, Pz, P4, P8, O1, Oz, and O2). Significant values were then examined in time-frequency diagrams (for an example, see Figure 2). Based on the inspection of these diagrams, we chose the following time segments for analysis: (1) 500 ms after the first metronome beat (i.e., the time interval between -750 and -250 ms) for the preparatory phase of tempo setting; (2) 500 ms each before and after both play onsets; (3) an additional 500-ms segment from a part of the Rondo where there was no play onset, extending from the second to the fifth second of the piece. Informed by earlier studies, our analyses were restricted to delta (1–4 Hz) and theta (4–8 Hz) frequency bands. Phase locking patterns were analyzed using a Four-Way repeated-measures ANOVA, testing the effects of musical role (leader vs. follower), electrode site (frontal, central, and parieto-occipital), frequency band (delta vs. theta) and time segment (six segments of 500 ms each: during preparatory tempo setting, before the first play onset, after the first play onset, during joint playing without play onset, before the second play onset and after the second play onset). For this analysis, PLI values were normalized using Fisher's z-transform and then averaged within the corresponding time intervals as well as frequency ranges. Greenhouse-Geisser epsilons were used for non-sphericity correction when necessary. Main effects and interactions with *p* < 0.05, that were relevant for the hypotheses of this study, were followed up by paired-samples *T*-tests comparing specific conditions. Follow-up results reported in the Results section were statistically significant at *p* < 0.05, with Bonferroni corrections when appropriate. To enhance readability of the Results section, the test statistics of the reported comparisons are shown in Appendix tables in the Appendix B.

#### Graph analysis and statistical evaluation of intra- and interbrain phase coherence

**Figure 3:**
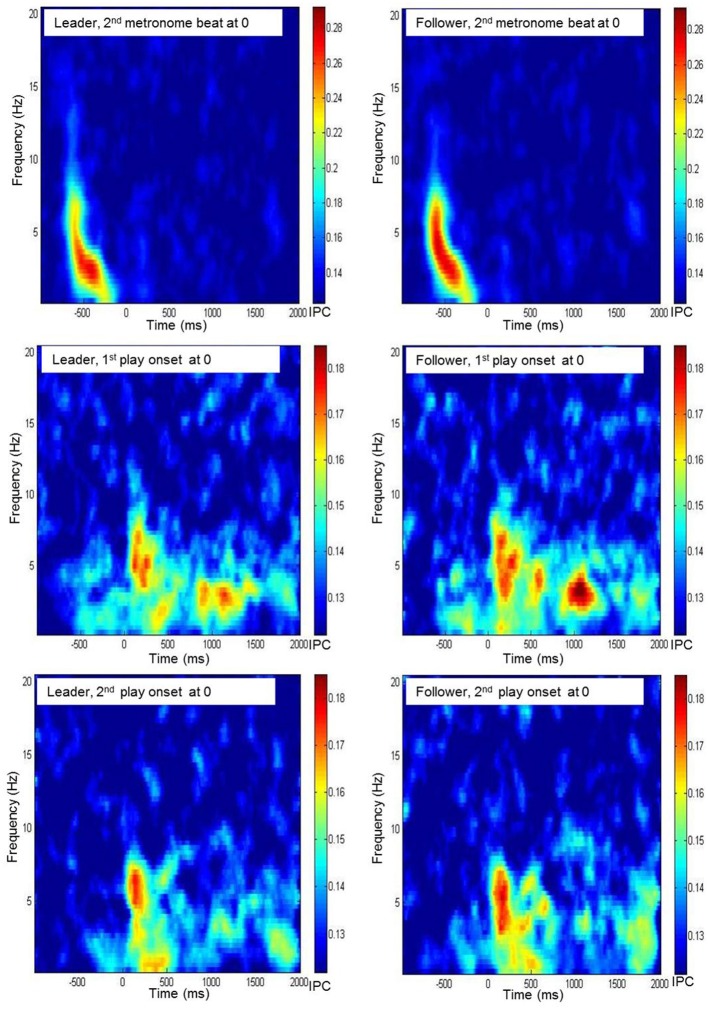
**Time frequency diagrams of the grand average of interbrain phase coherence, averaged across all electrode pairs of Fz of the leader's resp. the follower's brain with any electrode of the partner's brain during preparatory tempo setting and around coordinated play onsets**.

As for the PLI values, significance threshold for phase coherence measures was computed by means of surrogate data. To this end, we calculated the phase coherence between all pairs of shuffled EEG time series, and drew 1000 bootstrapping samples from the coherence values. Again, the threshold was defined as *M*_boot_ + 3×SD_boot_. This resulted in the same critical value as for PLI, i.e., 0.12. As before, data were inspected in time-frequency diagrams by averaging phase coherence values across all electrode pairs related to three reference electrodes, Fz, Cz, and Pz (see Figure 3 for an example). Except for the 500 ms before the two play onsets, for which no foci for phase coherence were found, we looked at the same time segments as for PLI, and again restricted our statistical analyses to the delta and theta frequency bands.

**Figure 4:**
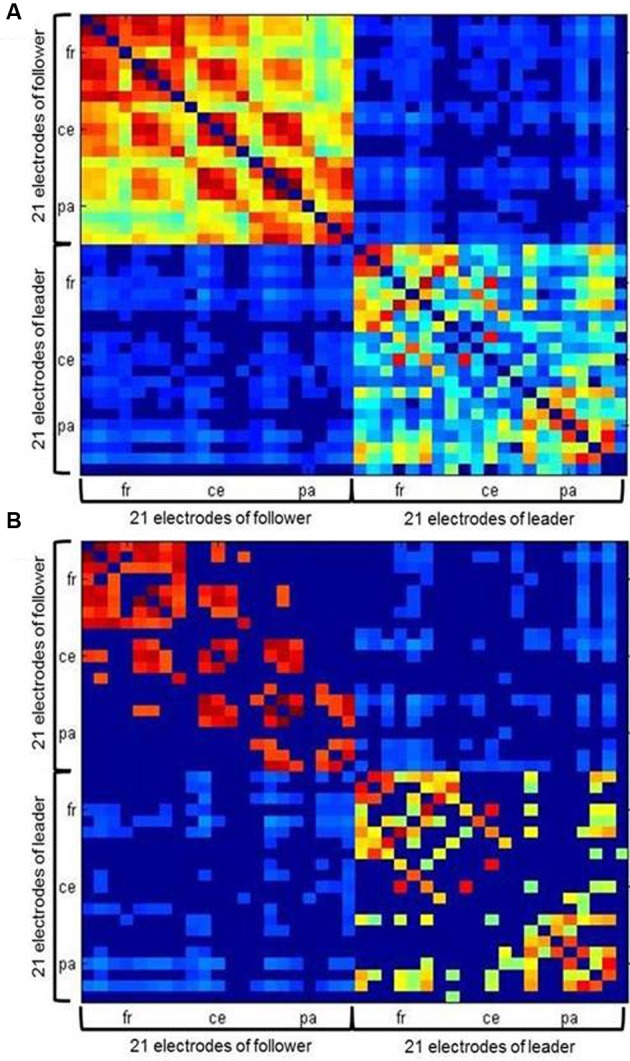
**Example of a hyperbrain network with (A) an absolute threshold of 0.12 and (B) an additional proportional threshold of 30 percent applied separately for within- and between brain connections**. Within-brain coherence of the follower is captured in the upper left, within-brain coherence of the leader in the lower right. Between-brain coherence is shown in the upper right and lower left of the matrix. The auto-coherence on the main diagonal is set to zero. For each interaction partner, 21 electrodes are arranged in the following order: Fp1, Fpz, Fp2, F7, F3, Fz, F4, F8, T7, C3, Cz, C4, T8, P7, P3, Pz, P4, P8, O1, Oz, and O2 from top (follower) to bottom (leader) and left (follower) to right (leader).

**Figure 5:**
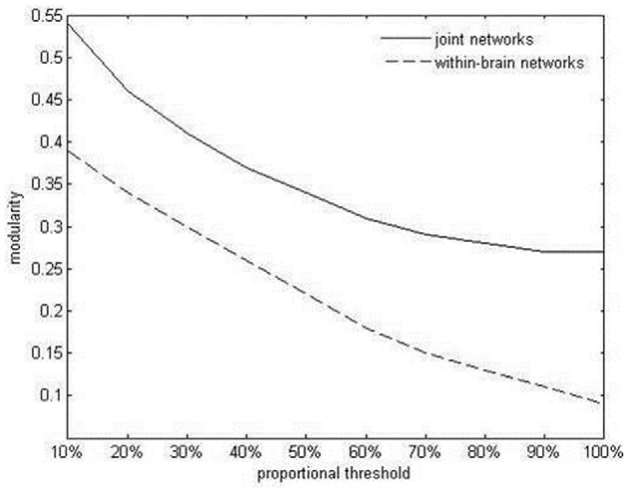
**Average within-brain and hyperbrain network modularity in the delta band, after the first play onset, as a function of thresholding**.

Values of intrabrain and IPC were combined into symmetrical coherence matrices containing all 42 electrodes from both duet partners (see Figure 4A for an example). These functional hyperbrain networks formed the basis of a graph analysis, which was conducted using the Brain Connectivity Toolbox developed by Rubinov and Sporns (2010). A proportional threshold was applied separately to the within- and the between-brain part of the matrix, leaving in only the strongest 30% of the within- and between-brain connections, respectively (see Figure 4B). To determine this threshold we took the delta network in the segment after the first play onset as a proxy, tentatively applied ten possible thresholds between 10 and 100% (10, 20, 30, …, 90, 100%, with 100 meaning no threshold) and plotted these against the resulting average modularity values for the within-brain and the hyperbrain networks (see Figure 5). The 30% threshold was chosen because it was the lowest threshold (i.e., the threshold least modifying the original networks) whose modularity values were close to or above the value of 0.3, which has been suggested as a lower boundary for non-random community structures (Newman and Girvan, 2004; Meunier et al., 2009).

The sum of weighted links connected to a node, termed strength, was calculated as indicators of a node's (electrode's) importance within the network. This was done for the hyperbrain as well as for the within-brain networks. Strengths for the between-brain partition of the hyperbrain networks were derived by subtracting the within-brain strengths from the hyperbrain strengths. Effects of role (leader vs. follower), time segment (four segments of 500 ms each: during preparatory tempo setting, after the first play onset, during joint playing without play onset and after the second play onset), electrode site (frontal, central, parieto-occipital; see “Statistical evaluation of phase-locking values”) and frequency bands (delta vs. theta) on hyperbrain, within- and between-brain strengths were evaluated using a Four-Way repeated-measures ANOVA.

To indicate the degree of functional integration, the characteristic path length (CPL), that is, the average shortest path length between all pairs of nodes in the network was determined separately for hyperbrain and within-brain networks. As a measure of functional segregation, we calculated hyperbrain and within-brain clustering coefficients (CC), that is, the fraction of a node's neighbors that are also neighbors of each other. Small-world networks are characterized by the simultaneous presence of functional integration and segregation, as indexed by relatively low values for CPL and relatively high values for CC, respectively. For within-brain analyses, both CPL and CC were examined using Three-Way repeated measures ANOVAs with role, segment and frequency as factors. We did not consider regional subdivisions here, as CPL is a global network property (Watts and Strogatz, 1998). For CPL and CC in the hyperbrain networks, Two-Way repeated-measures ANOVAs testing effects of segment and frequency were computed. The effect of role could not be analyzed for the functional interbrain connectivity, since the IPC is an undirected measure, which means that the connection from electrode A to electrode B has the same IPC value than the connection from B to A. The hyperbrain networks were accordingly symmetric and the between-brain connections were therefore identical for leader and follower. As for the PLI analyses, for the ANOVAs of the aforementioned graph analytical measures, main effects and interactions with *p* < 0.05 relevant for the hypotheses were followed up by pairwise comparisons. Follow-up results reported in the Results section were again statistically significant at *p* < 0.05, with Bonferroni corrections when appropriate. The test statistics of the reported follow-up comparisons are accordingly shown in Appendix tables in the Appendix B.

To further explore the structural properties of the networks, we also computed the degree of within-brain and hyperbrain network modularity, that is, the extent to which the network can be subdivided into non-overlapping groups of nodes with a maximal number of within-group links and a minimal number of between group links. Inspection of the data did not suggest substantial differences between playing conditions or frequency bands. Hence, we restricted the identification of the exact modular structure, which puts high demands on computing time, to the segment after the first play onset and the delta band.

## Results

### Phase locking index (PLI)

A Four-Way repeated measures ANOVA (role × segment × site × frequency) showed main effects of role, *F*_(1, 23)_ = 9.87, *p* = 0.005, η^2^_*p*_ = 0.30, segment, *F*_(2.18, 5.14)_ = 57.22, *p* < 0.001, η^2^_*p*_ = 0.71, site, *F*_(1.60, 36.75)_ = 38.45, *p* < 0.001, η^2^_*p*_ = 0.68, and frequency, *F*_(1, 23)_ = 92.54, *p* < 0.001, η^2^_*p*_ = 0.80. In general, phase locking was higher (1) in leaders than in followers, (2) during preparatory tempo setting, before and after play onsets than during joint playing without play onsets and higher after than before the play onsets (see Appendix Table B1), (3) in frontal and central than in parietal electrodes (see Appendix Table B2), and (4) for delta than theta.

**Figure 6:**
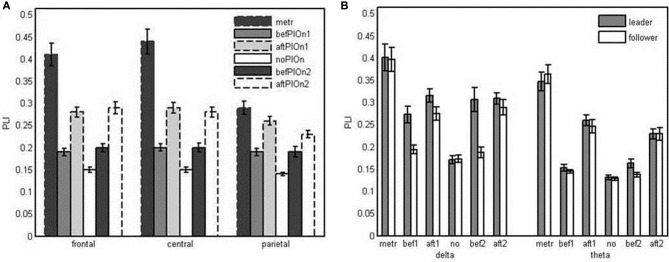
**(A)** Bar plot presentation of the interaction of segment and site in the four-way repeated measures ANOVA of Phase Locking Index (PLI). metr = 500 ms during preparatory tempo setting (after the first metronome beat); bef/aftPlOn1 = 500 ms before/after the first play onset of the leading guitarist; noPlOn = 500 ms of joint playing without play onset; bef/aftPlOn2 = 500 ms before/after the second play onset of the leading guitarist. **(B)** Bar plot presentation of the interaction of role and segment in the simple-effects ANOVAs of Phase Locking Index (PLI) for delta and theta frequencies, respectively. metr = 500 ms during preparatory tempo setting (after the first metronome beat); bef/aft1 = 500 ms before/after the first play onset of the leading guitarist; no = 500 ms of joint playing without play onset; bef/aft2 = 500 ms before/after the second play onset of the leading guitarist.

In addition, we observed a significant interaction of segment and site, *F*_(4.43, 101.92)_ = 27.24, *p* < 0.001, η^2^_*p*_ = 0.54. At all electrode sites, phase locking was significantly enhanced during preparatory tempo setting and after both play onsets relative to playing without onset. This enhancement was especially pronounced at frontal and central electrodes. Thus, relative to playing without onset, frontal and central electrodes showed stronger phase locking than parietal and occipital electrodes during preparatory tempo setting and after both play onsets. Phase locking was significantly higher after than before play onsets at all electrode sites, except for parietal electrodes after the second play onset (see Figure 6A and Appendix Table B3).

The three-way interaction of role, segment and frequency also was statistically significant, *F*_(5, 115)_ = 5.10, *p* < 0.001, η_p^2^= 0.18_. Follow-up analyses confirmed the interaction between role and segment for both delta, *F*_(3.28, 75.52)_ = 6.59, *p* < 0.001, η^2^_*p*_ = 0.21, and theta, *F*_(3.43, 78.87)_ = 3.93, *p* < 0.01, η^2^_*p*_ = 0.15. In the delta frequency band, the leaders' phase locking was increased relative to no play onset during preparatory tempo setting as well as before and after the play onsets. In contrast, followers did not show increased phase locking before the play onsets, resulting in a reliable difference in delta phase locking between leaders and followers both before the first play onset and before the second play onset. Before the second play onset, leaders also showed higher theta phase locking than followers (see Figure 6B and Appendix Table B4).

### Graph analysis of intra- and interbrain phase coherence

#### Strengths

Node strengths of the within-brain only, between-brain only, and hyperbrain (i.e., the conjunction of within- and between-brain) networks were evaluated in three separate Four-Way repeated measures ANOVAs (role × segment × site × frequency).

**Figure 7:**
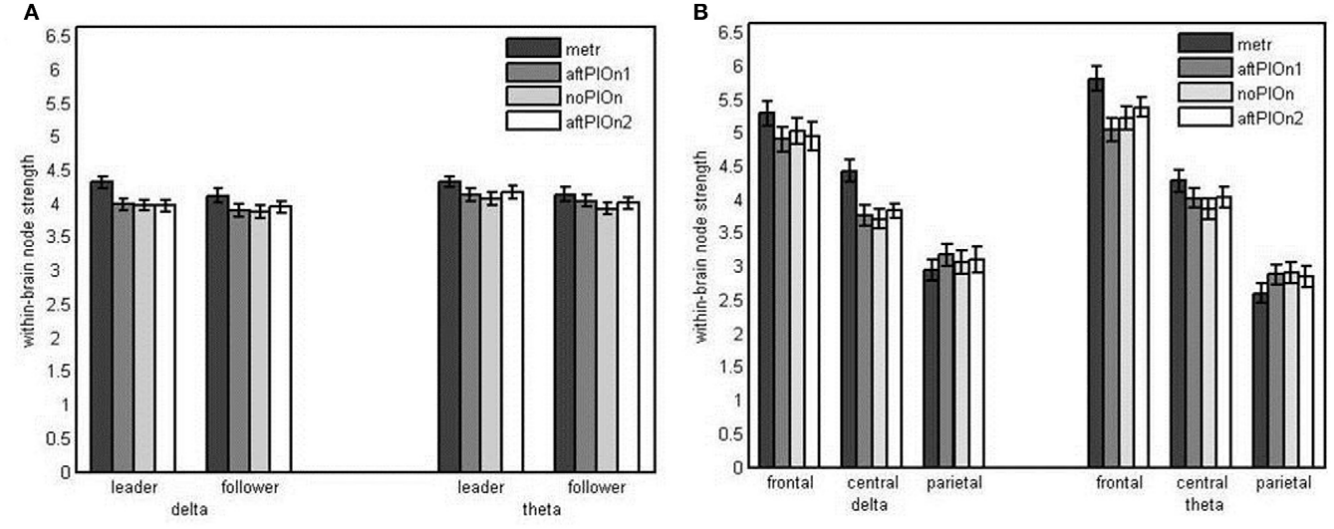
**(A)** Bar plot presentation of the interaction of role and segment in the simple-effects ANOVA of within-brain node strengths in delta frequencies (for theta frequencies, the interaction was not significant). **(B)** Bar plot presentation of the interaction of segment and site in the simple-effects ANOVAs of within-brain node strengths in delta and theta frequencies, respectively *Note*: metr = 500 ms during preparatory tempo setting (after the first metronome beat); bef/aftPlOn1 = 500 ms before/after the first play onset of the leading guitarist; noPlOn = 500 ms of joint playing without play onset; bef/aftPlOn2 = 500 ms before/after the second play onset of the leading guitarist.

***Node strengths analysis of within-brain networks*** We found main effects of segment, *F*_(1.75, 4.31)_ = 22.40, *p* < 0.001, η^2^_*p*_ = 0.49, site, *F*_(1.36, 31.17)_ = 48.33, *p* < 0.001, η^2^_*p*_ = 0.68, and frequency, *F*_(1, 23)_ = 7.17, *p* < 0.05, η^2^_*p*_ = 0.24. Within-brain node strengths were greater (a) during preparatory tempo setting than during playing without play onset (see Appendix Table B1), (b) at frontal than at central and higher at central than at parietal electrodes (see Appendix Table B2), and (c) for theta than delta. These main effects were qualified by three-way interactions of role, segment and frequency, *F*_(3, 69)_ = 3.05, *p* < 0.05, η^2^_*p*_ = 0.12, as well as segment, site and frequency, *F*_(2.70, 62.15)_ = 3.22, *p* < 0.05, η^2^_*p*_ = 0.12. Follow-up analyses showed that the interaction between role and segment was restricted to delta, *F*_(3, 69)_ = 4.41, *p* < 0.01, η^2^_*p*_ = 0.16. Both leaders' and followers' node strengths in delta were greater during the preparatory tempo setting than during joint playing without play onset. However, this difference was more pronounced in leaders (see Figure 7A and Appendix Table B5). The interaction between segment and site was present for delta, *F*_(2.87, 66.11)_ = 6.80, *p* = 0.001, η^2^_*p*_ = 0.23, and theta, *F*_(2.80, 64.43)_ = 8.29, *p* < 0.001, η^2^_*p*_ = 0.27. In delta, only central sites showed greater strengths during the preparatory tempo setting than during joint playing without play onset. In theta, this effect was present at both central and frontal sites (see Figure 7B and Appendix Table B6).

**Figure 8:**
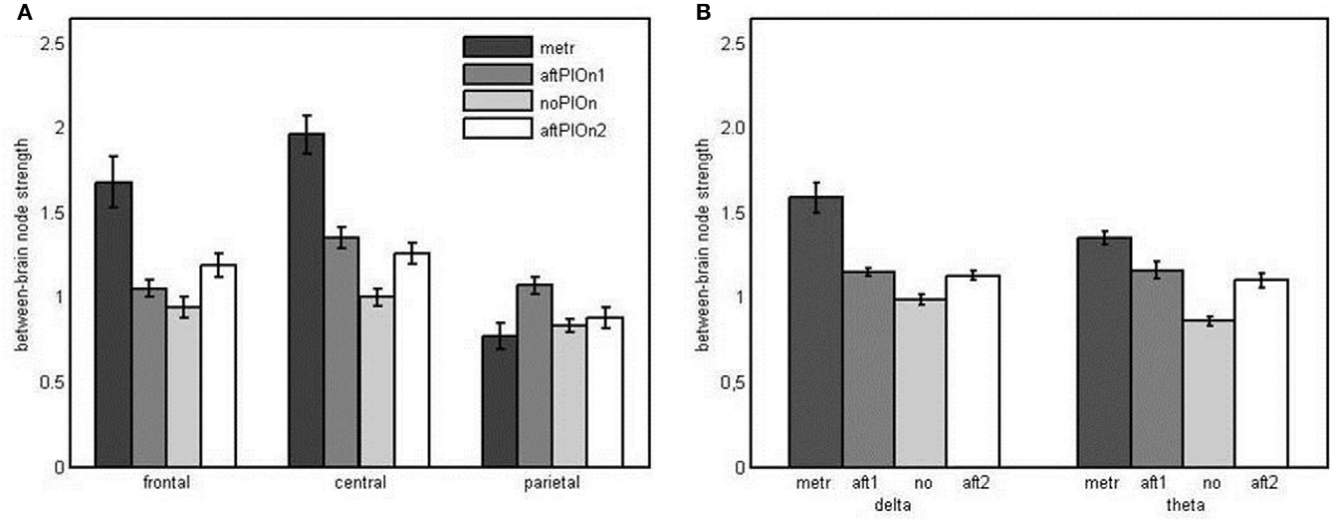
**(A)** Bar plot presentation of the interaction of segment × site in the four-way repeated measures ANOVA of node strengths in the between-brain network. metr = 500 ms during preparatory tempo setting (after the first metronome beat); bef/aftPlOn1 = 500 ms before/after the first play onset of the leading guitarist; noPlOn = 500 ms of joint playing without play onset; bef/aftPlOn2 = 500 ms before/after the second play onset of the leading guitarist. **(B)** Bar plot presentation of the interaction of segment and frequency in the four-way repeated measures ANOVA of node strengths in the hyperbrain network. metr = 500 ms during preparatory tempo setting (after the first metronome beat); bef/aft1 = 500 ms before/after the first play onset of the leading guitarist; no = 500 ms of joint playing without play onset; bef/aft2 = 500 ms before/after the second play onset of the leading guitarist.

***Node strengths analysis of between-brain networks*** Here, we found main effects of segment, *F*_(1.36, 31.19)_ = 47.85, *p* < 0.001, η^2^_*p*_ = 0.68, site, *F*_(1.50, 34.89)_ = 28.78, *p* < 0.001, η^2^_*p*_ = 0.56, and frequency, *F*_(1, 23)_ = 14.57, *p* = 0.001, η^2^_*p*_ = 0.39. Generally, node strengths were greater (a) during the preparatory tempo setting as well as after the play onsets than during joint playing without play onset (see Appendix Table B1), (b) at central sites than at other sites (see Appendix Table B2), and (c) in the delta band than in the theta band. These observations were qualified by an interaction between segment and site, *F*_(3.38, 77.78)_ = 13.70, *p* < 0.001, η^2^_*p*_ = 0.37, indicating that at node strengths were greater during the preparatory tempo setting and after both play onsets than during joint playing without play onset at central sites, while they were only greater during preparatory tempo setting and after the second play onset at frontal sites. At parietal sites, strengths were only greater after the first play onset than during joint playing without play onset (Figure 8A and Appendix Table B3). Additionally, there was a two-way interaction for segment and frequency, *F*_(2.01, 46.29)_ = 8.15, *p* = 0.001, η^2^_*p*_ = 0.26. Follow-up analyses showed that strengths were higher during the preparatory tempo setting and after the play onsets than during joint playing without play onset in both delta and theta. In delta, however, the difference between strengths during preparatory tempo setting and during joint playing without play onset was more pronounced (see Figure 8B and Appendix Table B7).

**Figure 9:**
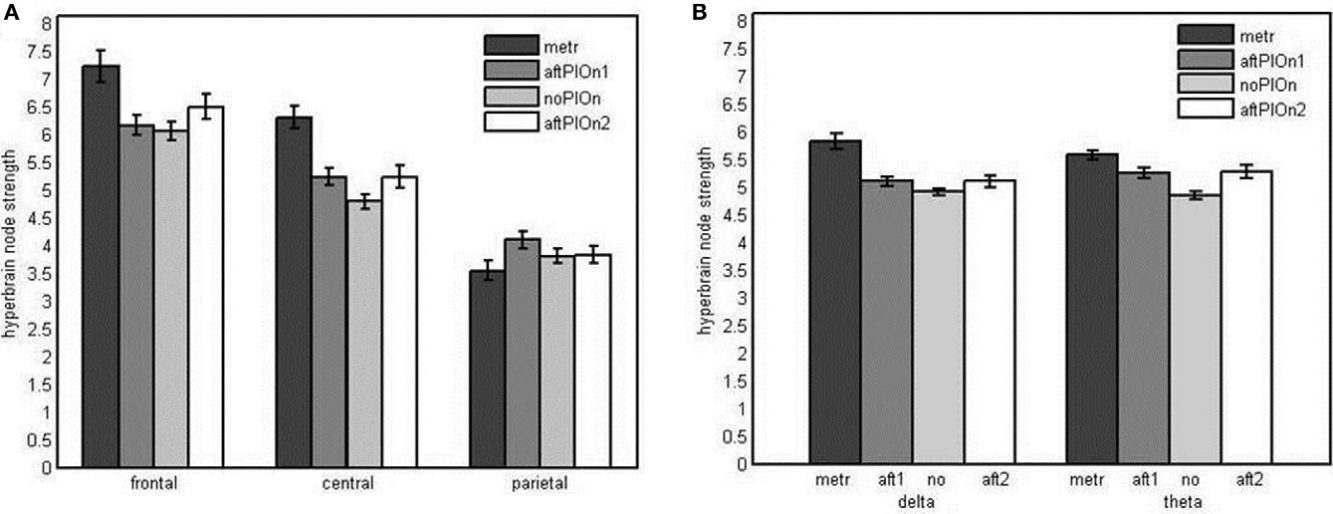
**(A)** Bar plot presentation of the interaction of segment and site in the four-way repeated measures ANOVA of node strengths in the hyperbrain network. **(B)** Bar plot presentation of the interaction of segment and frequency in the Four-Way repeated measures ANOVA of node strengths in the hyperbrain network *Note*: metr = 500 ms during preparatory tempo setting (after the first metronome beat); bef/aftPlOn1 = 500 ms before/after the first play onset of the leading guitarist; noPlOn = 500 ms of joint playing without play onset; bef/aftPlOn2 = 500 ms before/after the second play onset of the leading guitarist.

***Node strengths analysis of hyperbrain networks*** We observed main effects of segment, *F*_(1.45, 33.24)_ = 46.39, *p* < 0.001, η^2^_*p*_ = 0.67, and site, *F*_(1.37, 31.49)_ = 56.96, *p* < 0.001, η^2^_*p*_ = 0.71. Node strengths in hyperbrain networks were generally greater during preparatory tempo setting and after the play onsets relative to joint playing without play onsets (see Appendix Table B1). Frontal sites showed greater strengths than central as well as parietal sites and central sites showed greater strengths than parietal sites (see Appendix Table B2). The two main effects were qualified by an interaction between segment and site, *F*_(2.94, 67.67)_ = 15.92, *p* < 0.001, η^2^_*p*_ = 0.41. Follow-up tests revealed that central electrodes showed higher strengths during preparatory tempo setting and after the first play onset relative to joint playing without play onset, while frontal electrodes showed higher strengths during preparatory tempo setting only (see Figure 9A and Appendix Table B3). The interaction between segment and frequency, *F*_(2.03, 46.71)_ = 11.74, *p* < 0.001, η^2^_*p*_ = 0.34, indicated that strengths were higher during preparatory tempo setting and after both play onsets relative to playing without play onset in theta; for delta, node strengths were only greater during the preparatory tempo setting relative to playing without play onset (see Figure 9B and Appendix Table B7).

#### Small-worldness

**Figure 10:**
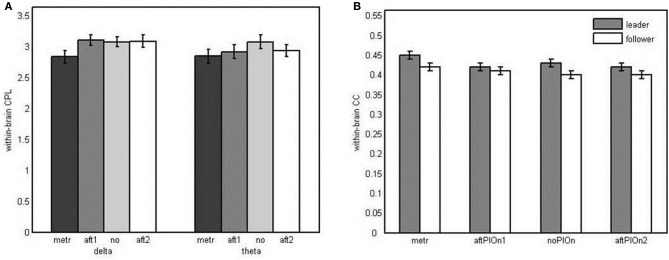
**(A)** Bar plot presentation of the interaction of segment and frequency in the three-way repeated measures ANOVA of characteristic path lengths in the within-brain network. **(B)** Bar plot presentation of the interaction of role and segment in the three-way repeated measures ANOVA of clustering coefficient in the within-brain network *Note*: metr = 500 ms during preparatory tempo setting (after the first metronome beat); bef/aftPlOn1 = 500 ms before/after the first play onset of the leading guitarist; noPlOn = 500 ms of joint playing without play onset; bef/aftPlOn2 = 500 ms before/after the second play onset of the leading guitarist.

***Small-worldness of within-brain networks*** CPL and CC were analyzed using Three-Way repeated measures ANOVAs (role × segment × frequency). Regarding CPL, we observed a main effect of segment, *F*_(2.12, 48.86)_ = 3.88, *p* < 0.05, η^2^_*p*_ = 0.14, and an interaction between segment and frequency, *F*_(3, 69)_ = 2.89, *p* < 0.05, η^2^_*p*_ = 0.11. The average shortest path length in the within-brain networks was shorter during the preparatory tempo setting than during joint playing without play onset (see Appendix Table B1), and the course of the CPL over the different time segments was modulated by frequency (see Figure 10A). The predicted data pattern—lower CPL at metronome beats and after the play onsets relative to joint playing without onset—seemed present in the theta frequency band, but the results of the corresponding follow-up tests were not statistically significant after Bonferroni correction. With regard to CC, we observed a main effect of segment, *F*_(3, 63)_ = 9.24, *p* < 0.001, η^2^_*p*_ = 0.31, indicating stronger clustering during the preparatory tempo setting than during joint playing without play onsets again (see Appendix Table B1). Furthermore, the interaction of role and segment, *F*_(3, 63)_ = 3.23, *p* < 0.05, η^2^_*p*_ = 0.13, indicated a differential course of within-brain clustering for leaders vs. followers (see Figure 10B). However, the corresponding follow-up analyses did again not yield reliable effects. Nevertheless, the main effects of segment indicate small world properties of the within-brain networks during preparatory tempo setting.

***Small-worldness of hyperbrain networks*** For CPL, we found a significant main effect of frequency, *F*_(1, 23)_ = 6.31, *p* < 0.05, η^2^_*p*_ = 0.22, indicating shorter path lengths for the delta than for the theta band. We also observed a main effect of segment, *F*_(2.27, 52.11)_ = 19.32, *p* < 0.001, η^2^_*p*_ = 0.46, due to shorter path lengths during the preparatory tempo setting and after both play onsets than during playing with no play onset (see Appendix Table B1). For CC, a main effect of segment was found, *F*_(2.04, 46.82)_ = 43.54, *p* < 0.001, η^2^_*p*_ = 0.65: CC was higher during the preparatory tempo setting and after the first coordinated play onset than during playing without play onset (see Appendix Table B1). Thus, small-world characteristics of the hyperbrain networks were observed during preparatory tempo setting and after the first play onset.

#### Community structure after the first play onset (delta band)

***Modular structure of within-brain brain networks*** The average modularity of the within-brain functional networks in the 500 ms after the first play onset was.3 (SD = 0.08) for followers and 0.29 (SD = 0.10) for leaders. Thus, the leaders' networks just missed the threshold of non-random community structures proposed by Newman and Girvan (2004); Meunier et al. (2009). Nevertheless, we took a closer look at them and found that the within-brain networks of both, leaders and followers, typically formed two modules, with a range of up to three in leaders and up to five in one single follower. One module was generally anterior (prefrontal/frontal), and the other generally posterior (parietal/occipital), with central and temporal electrodes being present in both. It was more commonly observed in leaders rather than followers that modules contained both frontal and parietal/occipital electrodes.

**Figure 11:**
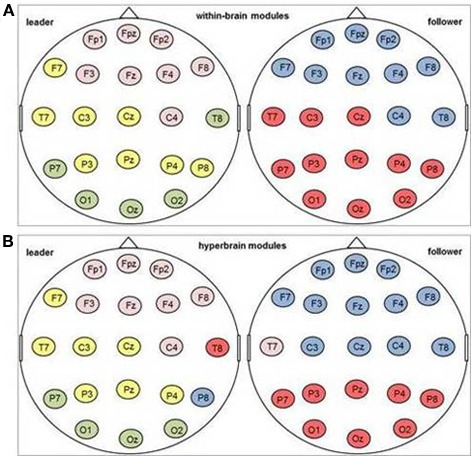
**One example for a modular community structure in the delta frequency band after the first coordinated play onset**. Electrodes marked in the same color belong to one module. **(A)** Modules in the within-brain network; **(B)** modules in the hyperbrain network.

***Modular structure of hyperbrain networks*** The average modularity of the hyperbrain networks was 0.41 (SD = 0.04), which implies a non-random community structure (Newman and Girvan, 2004; Meunier et al., 2009). The 42 electrodes of the hyperbrain networks were grouped in 3–6 modules, with a modal value of four. On average, two thirds of the modules of a given hyperbrain network comprised electrodes from both brains. Typically, these hyperbrain modules were composed of many electrodes of one brain and only a few of the other. A closer look indicated that the brain with the larger number of electrodes was primarily represented by frontal or parietal electrodes, whereas the brain with the smaller number of electrodes was primarily represented by temporal electrodes. Patterns differentiating between the two musical roles were not easily discernible. Figure 11 shows two typical examples of the modular structure of the within-brain (see Figure 11A) and the total brain network (see Figure 11B) as well as the corresponding patterns of intra- and interbrain connections.

## Discussion

### Summary and interpretation of findings

As noted by Lindenberger et al. (2009), similarities in brain oscillations observed between two or more individuals engaged in joint action may reflect, to a large degree, similarities in perceptual input and motor output between the interaction partners, rather than brain mechanisms at the service of IAC. In this study, we took several measures to attenuate this problem. In contrast to the work of Lindenberger et al. (2009), in which pairs of guitarists were playing in unison, guitarists in the present study were playing in two voices, thereby reducing similarities in movement, proprioception, and perception. Informed by Lindenberger et al. (2009), we predicted that phase synchronization within and between players would be most pronounced during preparatory tempo setting and at coordinated play onsets, when the need to coordinate is particularly high. Finally, we experimentally assigned the musical roles of leader and follower to each guitarist, and predicted that synchronization patterns would vary as a function of role assignment.

We examined the degree of phase locking at single electrodes as well as phase coherence between pairs of electrodes within one brain and between two brains. As predicted, phase locking, within- and between-brain phase coherence were enhanced during preparatory tempo setting and during musical coordination periods, especially at frontal and central electrode sites. This finding extends the results of Lindenberger et al. (2009) to a situation in which action and perception differ between interaction partners. The prominent role of fronto-central electrode sites is consistent with the assumption that the representation of one's owns and the other person's actions in real time and their partial integration into a joint, interpersonally shared forward model may help to initiate and sustain IAC.

We also found that phase locking was modulated in relation to the musical roles of leader and follower. Leaders generally showed higher phase locking than followers. Furthermore, phase locking followed a different time course in leaders vs. followers: While delta phase locking started only after the play onsets in followers, it set in already before coordinated play onset in leaders, resulting in significantly higher phase locking in leaders than in followers at this early point in time. This difference may reflect the decision on the part of the leader to initiate playing (Basar-Eroglu et al., 1992). Only before the second play onset did the higher phase locking in leaders also extend to theta frequencies. Note that the second play onset was characterized by faster tempo after the preceding decrease in tempo; hence, one may speculate that higher musical tempo was reflected in phase locking at higher frequencies (cf. Lindenberger et al., 2009). This interpretation is consistent with studies suggesting that response-preceding synchronization reflects accurate timing in selective attention (cf. Delorme et al., 2007).

To investigate phase coherence within and between brains in greater detail, we applied methods from graph theory to examine node strengths, small-world properties, and community structures in within-brain and hyperbrain networks.

In line with our hypotheses, we found that node strengths, defined as the sum of weighted links connected to a node, were greater during musical coordination periods than during joint playing without play onset in the within-brain only, the between-brain only and in the hyperbrain networks. Also, frontal and central electrodes were more important than parietal electrodes in all three types of networks we examined. This finding again confirms the prominent role of frontal and central areas in interpersonal action coordination (Sänger et al., 2011). Differently from the within-brain networks, frontal and central electrodes showed higher strengths not only during preparatory tempo setting, but also after the coordinated play onsets in the between-brain networks. This observation is in line with a study by Zatorre et al. (2007) who showed that music production draws on sensorimotor areas. It also supports our conceptual model (Sänger et al., 2011), according to which individuals engaged in joint action with high demands on real-time coordination represent both their own actions and the actions of their partners as forward models implemented in an oscillatory neural code.

In the between-brain partition of the hyperbrain networks, delta strengths were generally higher than theta strengths, while theta strengths were higher in the within-brain partition of the hyperbrain network. Apparently, then, intrabrain synchronization operated at faster frequencies than interbrain synchronization. Future analyses need to explore n:m couplings (e.g., Von Stein and Sarnthein, 2000) between delta and theta frequency bands and their frequency dependence on the metrum of the music (e.g., Lindenberger et al., 2009).

Next, we computed CPL and CC to capture the small-world properties of hyperbrain networks and their within-brain partitions. The small-world properties of hyperbrain networks, as indexed by relatively low values for CPL and relatively high values for CC, were enhanced during preparatory tempo setting and after the first play onset. In within-brain partitions, this effect was restricted to preparatory tempo setting. Taken together, these results are consistent with the hypothesis that small-world network characteristics of brain networks are present during IAC, and then enhanced during time periods that impose particularly high coordination demands. Small-world properties optimize complexity and facilitate communication (Sporns and Zwi, 2004). Tononi and Edelman (1998) discuss this network property as a possible correlate of decision making and planning, and as a putative neural basis of consciousness. It may thus be worth further exploring whether small-world properties of hyperbrain networks during joint action are accompanied by the subjective experience of “feeling in synch” with the interaction partner.

When examining the community structure of delta oscillations after the first play onset, we observed non-random community structures for hyperbrain networks and marginally non-random community structures for within-brain networks, according to criteria proposed by Newman and Girvan (2004) and Meunier et al., 2009. The networks of leaders were more likely to contain modules comprised of electrodes from distant parts of the brain than the networks of followers, perhaps reflecting different cognitive states associated with different network structures (cf. Schutter and Van Honck, 2004). Dehaene et al. (1998) has linked a network of distributed and interconnected neural ensembles with the notion of a “global workspace,” which is activated by effortful tasks. Hence, the observed differences in within-brain networks may reflect that the role of the leader is associated with greater effort than the role of the follower.

The hyperbrain networks of the various pairs were grouped into two up to six modules, with a modal value of four. Two thirds of the modules identified were hyperbrain modules, that is, they contained electrodes from both brains. Hyperbrain modules were typically composed of a big cluster of frontal or parietal electrodes from one brain, and only few (fronto-/parieto-) temporal electrodes from the other brain. This finding is in line with results by Lindenberger et al. (2009), who observed interbrain synchronization involving temporal and parietal regions. Regions at the parieto-temporal boundary have been found to play a crucial role in mapping auditory representations onto motor representations of melodies (Hickok et al., 2003), which may be an important process in joint music production. In addition, parietal regions have been associated with social cognitive functions such as agency (Decety et al., 2002, 2004) that also seem relevant in IAC. In line with the phase locking and phase coherences findings reported above, the involvement of larger numbers of fronto-central electrodes from the other brain may represent coordinated firing of neuronal assemblies located in motor and somatosensory cortex. Besides their functions in motor activity, such assemblies have been linked to social cognition, in general, and theory of mind abilities, in particular (Rizzolatti et al., 2001; Gallagher and Frith, 2003). In sum, it seems that the hyperbrain modules identified in this study may connect areas from two different brains that have been associated with social cognition and music production. Clearly, this conjecture needs to be corroborated by further research.

### Limitations and open questions

Research on neural correlates of IAC is still in its beginnings. Hence, as is true for other innovative work, the results of this study are in need of replication, and should be interpreted cautiously. In the following, we focus on a select number of limitations and open issues.

First, despite the shift from unison playing (Lindenberger et al., 2009) to playing in two voices, the similarities in the dynamics of motor performance and perceptual input between two players remain substantial, and are likely to contribute to similarities in oscillatory patterns. Note, however, that this overall similarity does not offer a sufficient explanation for the pervasively observed increase in synchronous oscillatory activity during time periods that impose high demands on musical coordination, given that these periods do not differ in perceptual and motor similarity from other segments of the musical score. To better control for similarities in motor performance, future studies may focus on listeners, individuals playing different instruments, or periods during which one musician is playing and the other is not.

Second, our exploration of hyperbrain structures was limited by our measures and statistical procedures. In this sense, our analyses represent first steps into a field that still needs to develop a repertoire of appropriate methodological tools. The symmetric coherence measures used in this study prevented us from exploring directed functional connections between the two brains, and the network properties we observed are contingent upon the thresholding procedure. The application of thresholds has been recommended to confine the topology to substantial and interpretable connections (Rubinov and Sporns, 2010). However, any threshold is arbitrary and may distort network properties. In future work, it is preferable to use directed measures of connectivity and statistical procedures that ascertain the robustness of the results obtained without thresholding. Moreover, we acknowledge that future work on hyperbrain structures should make use of multipartite graphs to more adequately capture the partitioning of the hyperbrain network into within-brain and between-brain component matrices.

Third, the relatively low spatial resolution of EEG and the absence of a source analysis greatly limit the ability to draw inferences about the functional role of specific brain areas on the basis of the present study. For instance, most references to specific brain areas in this article borrow heavily from related fMRI work (Lee et al., 2009; Schippers et al., 2010; Stephens et al., 2010). At the same time, the potential of EEG data to provide information about the source of neural activity, especially if complemented by other imaging modalities, is greater than commonly assumed (Michel and He, 2011; Michel and Murray, 2011). Future analyses of the present and related data sets should exploit this potential to a greater extent, and future studies on IAC should combine different imaging modality to optimize both spatial and temporal resolution (e.g., Michel and He, 2011).

Finally, the present design, which focused on phase locking and phase coherence across repeated trials, should be complemented by designs that focus on associations between neural and behavioral synchrony in continuous streams of behavior, such as musical improvisation. In this context, it seems worthwhile to adopt the behavioral methodology developed to assess behavioral symmetry and symmetry breaking in dancing or dyadic conversation (Boker and Rotondo, 2002) to the musical domain, in combination with electrophysiological recordings. Also would the example of musical improvisation provide the opportunity to investigate an instance of IAC that incorporates spontaneous turn-taking, thereby coming closer to actual social interaction than our rather synthetic trial-based laboratory design of joint music production.

## Conclusion

We investigated neural correlates of IAC by examining pairs of guitarists repeatedly playing a duet in two voices. Within-brain phase locking as well as within-brain and between-brain phase-coherence connection strengths were enhanced at frontal and central electrodes during periods that put particularly high demands on musical coordination. Phase locking was modulated in relation to the experimentally assigned musical roles of leader and follower. Hyperbrain networks during music performance showed small-world properties that were enhanced during musical coordination periods, and community structures encompassing electrodes from both brains (hyperbrain modules). Taken together, the present results considerably strengthen the claim made by Lindenberger et al. (2009), that synchronous oscillations within and between brains play a functional role in music performance, and support the more general conjecture that brain mechanisms indexed by phase locking, phase coherence, and structural properties of within-brain and hyperbrain networks support IAC.

### Conflict of interest statement

The authors declare that the research was conducted in the absence of any commercial or financial relationships that could be construed as a potential conflict of interest.

## Appendix A

**Table A1:** **Matching of age and qualification aspects of guitarists A and B in the 12 duets of the final sample**.

	**Age**		**Experience**		**Conservatory**		**Work**		**Ensemble**	
	**A**	**B**	**A**	**B**	**A**	**B**	**A**	**B**	**A**	**B**
1	30	41	12	17	n	n	n	n	y	y
2	46	46	38	39	y	y	y	y	y	y
3	58	50	48	20	n	n	n	n	n	n
4	20	25	11	14	y	y	n	y	y	y
5	29	40	18	30	y	y	y	y	y	y
6	41	47	25	37	n	n	y	y	y	y
7	26	30	10	11	n	n	n	n	n	y
8	30	28	17	15	n	n	y	y	n	n
9	37	28	29	16	y	n	y	y	y	y
10	44	47	30	33	n	n	n	n	y	n
11	29	22	10	7	n	n	n	n	n	n
12	49	20	25	13	y	y	y	n	y	n
	mean difference = 7.92 (SD = 7.48)		mean difference = 7.92 (SD = 7.93)		11 out of 12 concordant		10 out of 12 concordant		9 out of 12 concordant	

Experience: years since participant started to play the guitar

Conservatory: whether participant has studied/is studying guitar (y) or not (n)

Work: whether participant works as guitarist (y) or not (n)

Ensemble: whether participant is currently a member of a music ensemble (y) or not (n).

## Appendix B

Appendix tables showing means (*M*), standard deviations (SD), *T* and *p* values of hypotheses-relevant, significant follow-up T tests for the repeated-measures ANOVAs of Phase Locking Index (PLI) as well as node strengths, Characteristic Path Length (CPL) and Clustering Coefficient (CC) of within-, between- and hyperbrain networks.

metr = 500 ms during preparatory tempo setting (after the second metronome beat)

befPlOn1/befPlOn2 = 500 ms before the first/second play onset of the leading guitarist

aftPlOn1/aftPlOn2 = 500 ms after the first play/second onset of the leading guitarist

noPlOn = 500 ms of joint playing without play onset

**Table B1:** **Main effects of segment**.

	***M***	**SD**		***M***	**SD**	***T(23)***	***p***
**PLI**							
noPlOn	0.15	0.02	metr	0.38	0.11	−10.46	<0.001
			befPlOn1	0.19	0.04	−4.89	<0.001
			aftPlOn1	0.27	0.05	−4.35	<0.001
			befPlOn2	0.20	0.05	−12.95	<0.001
			aftPlOn2	0.26	0.05	−12.36	<0.001
befPlOn1	0.19	0.04	aftPlOn1	0.27	0.05	−8.70	<0.001
befPlOn2	0.20	0.05	aftPlOn2	0.26	0.05	−6.27	<0.001
**WITHIN−BRAIN STRENGTHS**							
noPlOn	3.97	0.29	metr	4.22	0.32	−4.67	<0.001
**BETWEEN−BRAIN STRENGTHS**								
noPlOn	0.92	0.14	metr	1.45	0.30	7.93	<0.001
			aftPlOn1	1.15	0.14	8.25	<0.001
			aftPlOn2	1.11	0.13	−9.12	<0.001
**HYPERBRAIN STRENGTHS**							
noPlOn	4.90	0.28	metr	5.61	0.50	7.77	<0.001
			aftPlOn1	5.16	0.33	6.55	<0.001
			aftPlOn2	5.13	0.31	−6.76	<0.001
**WITHIN−BRAIN CPL**							
noPlOn	3.08	0.41	metr	2.85	0.49	−2.98	0.007
**WITHIN−BRAIN CC**							
noPlOn	0.41	0.04	metr	0.44	0.04	3.58	<0.002
**HYPERBRAIN CPL**							
noPlOn	6.21	0.54	metr	4.94	0.81	6.40	<0.001
			aftPlOn1	5.60	0.68	4.73	<0.001
			aftPlOn2	5.60	0.59	4.37	<0.001
**HYPERBRAIN CC**							
noPlOn	0.23	0.02	metr	0.29	0.04	−8.10	<0.001
			aftPlOn1	0.25	0.02	−4.52	<0.001

**Table B2:** **Main effects of site**.

	***M***	**SD**		***M***	**SD**	***T(23)***	***p***
**PLI**							
Frontal	0.25	0.04	Parietal	0.22	0.03	5.76	<0.001
Central	0.26	0.04	Parietal	0.22	0.03	7.76	<0.001
**WITHIN-BRAIN STRENGTHS**							
Frontal	5.24	0.81	Central	3.99	0.65	8.31	<0.001
			Parietal	2.94	0.71	7.75	<0.001
Central	3.99	0.65	Parietal	2.94	0.71	4.53	<0.001
**BETWEEN-BRAIN STRENGTHS**							
Frontal	1.22	0.28	Central	1.39	0.24	−3.46	0.002
			Parietal	0.88	0.18	3.88	0.001
Central	1.39	0.24	Parietal	0.88	0.18	8.05	<0.001
**HYPERBRAIN STRENGTHS**							
Frontal	6.45	0.92	Central	5.38	0.69	6.37	<0.001
			Parietal	3.83	0.70	8.32	<0.001
Central	5.38	0.69	Parietal	3.83	0.70	6.59	<0.001

**Table B3:** **Interactions of segment and site**.

		***M***	**SD**		***M***	**SD**	***T(23)***	***p***
**PLI**								
Frontal	noPlOn	0.15	0.03	metr	0.41	0.13	−9.47	<0.001
				aftPlOn1	0.28	0.06	−9.45	<0.001
				aftPlOn2	0.28	0.07	−10.04	<0.001
Central	noPlOn	0.15	0.03	metr	0.44	0.14	−10.41	<0.001
				befPlOn1	0.20	0.04	−4.66	<0.001
				befPlOn2	0.20	0.05	−4.35	<0.001
				aftPlOn1	0.29	0.06	−13.87	<0.001
				aftPlOn2	0.27	0.06	−10.84	<0.001
Parietal	noPlOn	0.14	0.02	metr	0.29	0.07	−9.12	<0.001
				aftPlOn1	0.26	0.05	−13.17	<0.001
				aftPlOn2	0.23	0.04	−11.77	<0.001
metr	Frontal	0.41	0.13	Parietal	0.29	0.07	6.65	<0.001
	Central	0.44	0.14	Parietal	0.29	0.07	9.53	<0.001
aftPlOn1	Central	0.29	0.06	Parietal	0.23	0.04	4.21	<0.001
aftPlOn2	Frontal	0.28	0.07	Parietal	0.23	0.04	4.11	<0.001
**BETWEEN−BRAIN STRENGTHS**								
Frontal	noPlOn	0.94	0.28	metr	1.68	0.72	4.98	<0.001
				aftPlOn2	1.19	0.35	−4.41	<0.001
Central	noPlOn	1.00	0.23	metr	1.96	0.54	7.66	<0.001
				aftPlOn1	1.35	0.28	6.66	<0.001
				aftPlOn2	1.26	0.31	−4.42	<0.001
Parietal	noPlOn	0.83	0.17	aftPlOn1	1.07	0.23	4.60	<0.001
**HYPERBRAIN STRENGTHS**								
Frontal	noPlOn	6.06	0.83	metr	7.23	1.38	−5.10	<0.001
Central	noPlOn	4.79	0.66	metr	6.31	1.05	−7.75	<0.001
				aftPlOn1	5.24	0.79	−5.37	<0.001

**Table B4:** **Interaction of role and segment in the simple-effects ANOVA of PLI in delta and theta**.

		***M***	**SD**		***M***	**SD**	***T(23)***	***p***
**DELTA**								
Leader	noPlOn	0.17	0.04	metr	0.40	0.15	−7.53	<0.001
				befPlOn1	0.27	0.09	−5.30	<0.001
				befPlOn2	0.31	0.13	−4.80	<0.001
				aftPlOn1	0.28	0.07	−8.45	<0.001
				aftPlOn2	0.30	0.09	−8.67	<0.001
**THETA**								
Leader	noPlOn	0.13	0.02	metr	0.37	0.12	−9.50	<0.001
				befPlOn1	0.19	0.06	−4.99	<0.001
				befPlOn2	0.22	0.07	−5.89	<0.001
				aftPlOn1	0.28	0.06	−12.96	<0.001
				aftPlOn2	0.25	0.05	−11.94	<0.001
Follower	noPlOn	0.13	0.02	befPlOn1	0.16	0.02	−5.84	<0.001

**Table B5:** **Interaction of role, segment and frequency in the simple-effects ANOVA of within-brain node strengths in delta**.

		***M***	**SD**		***M***	**SD**	***T(23)***	***p***
Leader	noPlOn	4.03	0.34	metr	4.30	0.38	6.04	<0.001
Follower	noPlOn	3.89	0.43	metr	4.08	0.49	3.58	0.002

**Table B6:** **Interaction of segment and site in the simple-effects ANOVAs of within-brain node strengths in delta and theta**.

		***M***	**SD**		***M***	**SD**	***T(23)***	***p***
**DELTA**								
Central	noPlOn	3.71	0.67	metr	4.43	0.79	5.92	<0.001
**THETA**								
Frontal	noPlon	5.22	0.83	metr	5.81	0.95	4.79	<0.001
Central	noPlOn	3.86	0.73	metr	4.28	0.78	4.14	<0.001

**Table B7:** **Interactions of segment and frequency**.

		***M***	**SD**		***M***	**SD**	***T(23)***	***p***
**BETWEEN-BRAIN STRENGTHS**								
Delta	noPlOn	0.97	0.15	metr	1.52	0.42	5.79	<0.001
				aftPlOn1	1.13	0.11	4.02	0.001
				aftPlOn2	1.11	0.11	−5.33	<0.001
theta	noPlOn	0.85	0.16	metr	1.29	0.18	9.15	<0.001
				aftPlOn1	1.13	0.21	8.61	<0.001
				aftPlOn2	1.07	0.15	−9.42	<0.001
**HYPERBRAIN STRENGTHS**								
Delta	noPlOn	4.91	0.32	metr	5.82	0.70	−6.58	<0.001
Theta	noPlOn	4.85	0.36	metr	5.58	0.43	−9.13	<0.001
				aftPlOn1	5.25	0.44	−7.75	<0.001
				aftPlOn2	5.27	0.59	−4.99	<0.001
